# Early life stress and perceived social isolation influence how children use value information to guide behavior

**DOI:** 10.1111/cdev.13727

**Published:** 2021-12-31

**Authors:** Karen E. Smith, Seth D. Pollak

**Affiliations:** ^1^ University of Wisconsin–Madison Madison Wisconsin USA

## Abstract

Learning the value of environmental signals and using that information to guide behavior is critical for survival. Stress in childhood may influence these processes, but how it does so is still unclear. This study examined how stressful event exposures and perceived social isolation affect the ability to learn value signals and use that information in 72 children (8–9 years; 29 girls; 65.3% White). Stressful event exposures and perceived social isolation did not influence how children learned value information. But, children with high stressful event exposures and perceived social isolation were worse at using that information. These data suggest alterations in how value information is used, rather than learned, may be one mechanism linking early experiences to later behaviors.

**Abbreviations**
ECG: electrocardiogram
FDR: false discovery rate
HLM: hierarchical linear modeling
IBI: inter‐beat interval
RT: response time
VAS: Visual Analogue Scale

Learning to attach value to cues in the environment, such as those signaling salient potential outcomes including reward, threat, or safety, is essential for adaptive decision making (Daw & Tobler, 2013; Padoa‐Schioppa & Assad, 2006). This type of learning facilitates a wide range of behaviors including decisions about which foods to eat, making choices that avoid injuries, and effectively navigating the social world (Debiec & Olsson, 2017; Knutson & Srirangarajan, 2019). In contrast, deficits in value learning are implicated in behavioral disruptions including aggression, anxiety, impulsivity, and risk taking (Galván, 2013; J. E. LeDoux et al., 2017; Wuensch et al., 2021). Here, we examine associations between exposure to stress, perceived social isolation, and children's ability to learn signals of value and then make use that information to guide their subsequent decision making and behavior.

Chronic or extreme stress early in development has emerged as a factor that has long‐term effects on an organism's ability to learn about value outcomes (Palacios‐Barrios & Hanson, 2019). Indeed, recent evidence suggests disrupted value learning is one mechanism linking stress in childhood and adolescence with later negative behavioral outcomes (Fareri & Tottenham, 2016; Herzberg & Gunnar, 2020). However, there are inconsistencies in the literature that preempt a clear understanding of the role of early life stress on these learning systems. For example, some studies report that children ages 8–16 years and young adults who have experienced early life stress demonstrate poor performance on reward learning tasks (Boecker et al., 2014; Hanson et al., 2015; Kasparek et al., 2020). Yet other studies find no effect of early life stress on children (ages 6–19 years) and young adult's reward learning (Boecker‐Schlier et al., 2016; Dennison et al., 2017; Gerin et al., 2017). Similarly, some reports indicate that children ages 6–18 years old with early stress exposure have disrupted threat learning (McLaughlin et al., 2016), whereas others studies demonstrated limited evidence for effects of stress on threat learning in children ages 4–7 years (Machlin et al., 2019), and still other studies have found that stress enhances such learning in children ages 7–16 years (Silvers et al., 2016). Thus, the relation between early life stress and value learning later in development remains unclear.

The present experiment addresses two gaps in prior research that may contribute to these divergent findings. One potential issue contributing to this divergence is that predictive value learning is often measured as a single process. However, it encompasses at least two distinct components. First, children must be able to effectively learn the probabilistic relations between initially neutral or unfamiliar stimuli and some form of signaled value based on an outcome (Kringelbach & Berridge, 2017; J. LeDoux & Daw, 2018; O’Doherty et al., 2017). For example, the child must make the connection that a particular stimulus is salient and associate it with reward, threat, safety, or punishment. Second, children must harness this newly acquired information to adaptively guide their behaviors—for example, by using this information to execute action to obtain rewards or avoid threats or punishment (Debiec & Olsson, 2017; Glimcher, 2011). Early life stress could be associated with disruptions in one or both of these components; however, these have not been disaggregated in extant research.

A second issue that may obfuscate understanding of the relation between early life stress and value learning concerns how childhood stress is conceptualized and measured. Research in the area of childhood stress generally relies upon methods that focus on identifying a small subset of events that occurred in a child's life. Yet, research with adults and non‐human animals indicates that whether an individual responds to a potentially stressful event as a stressor is shaped not only by the event, but by a plethora of other factors, including individuals’ perceptions of social isolation (McEwen & Akil, 2020; Smith & Pollak, 2021). Perceived social isolation, which refers to perceiving oneself to lack meaningful social relationships, has been linked to increased levels of perceived stress, increased sensitivity to cues of threat, and exacerbated psychological and physiological responses to laboratory stress (Brown et al., 2017; McHugh & Lawlor, 2013; Smith et al., 2020). While research has not directly tested how perceived social isolation influences children's responses to potentially stressful events, perceived social isolation is linked to both increased perceptions of stress and hypersensitivity to threat in adolescents and young adults ages 13–20 years old (Vanhalst et al., 2013, 2017). Together this suggests that perceived social isolation is relevant for understanding the effects of childhood stress on value learning. In particular, it indicates any effects of stressful event exposures on value learning may be more pronounced in children who also report high levels of social isolation.

In the current exploratory study, we tested the relation between stressful event exposures, perceived social isolation, and children's value learning. To determine whether stressful event exposures and perceived social isolation are differentially associated with the two components of value learning, we separately assessed children's ability to learn a contingent value relation as well as their ability to use those learned relations to inform their behavioral choices. We expected any effects of stressful event exposures on the two components of value learning to be most pronounced in children who also reported high levels of perceived social isolation. Finally, we used a variety of different reinforcer types (both rewards and threats) to ensure generalizability of the results to learning processes.

## METHOD

### Participants

We aimed for a sample size of 70 children, consistent with prior developmental research on similar topics (Gerin et al., 2017; Harms et al., 2018) and recommendations from power simulation studies for hierarchical linear models (Kerkhoff & Nussbeck, 2019). Our final sample was 72 eight‐ to nine‐year‐old children (29 girls; *M*_age_ = 8.43; *SD* = 0.50; race: 65.3% White Non‐Hispanic; 2.8% Asian; 9.7% Black or African American; 9.7% White Hispanic; 4.2% Hispanic; 4.2% Multi‐Racial; 4.2% Race indicated as other) recruited from a Midwestern city (2019–2020). We recruited children in this age range because this appears to be the earliest period when children reliably exhibit both appetitive and aversive conditioned learning (Gerin et al., 2017; Michalska et al., 2016). To capture a range of potentially stressful experiences, we did not target any particular type of stress exposure. Parents reported a median household income between $75,000–$99,999 and an average education level of 16.38 years (*SD* = 2.49; equivalent to a 4‐year college degree). Children provided verbal assent, and their parents provided written informed consent. Child participants received a toy prize and their parents received $25 for participation. This study was approved by the University of Wisconsin—Madison Institutional Review Board (IRB).

### Procedure

Children first completed a conditioned learning task to assess their ability to learn associations between previously neutral stimuli and valued outcomes. After the conditioned learning task, children completed an approach and avoidance task, to assess whether they used the information they learned in the conditioning task to guide their behavior. Children also completed the Matrix Reasoning and Vocabulary subtests of the Wechsler Abbreviated Scale of Intelligence‐second edition (Wechsler, 2011) to account for individual differences in cognitive functioning and the Multidimensional Anxiety Scale for Children (March et al., 1997) and the Child Depression Inventory (Kovacs, 1985) to account for symptoms of depression and anxiety. To rule out the possibility that potential differences in performance between the two tasks were driven by differences in memory for the learned relations, participants completed an explicit recall task after the conditioning task and prior to the behavioral choice task. Parents reported their child's exposure to stressful life events, and children reported their own perceptions of social isolation. Tasks were presented using E‐Prime 2.0 on a touch screen Windows PC. An electrocardiogram (ECG) was collected using a standard lead II electrode configuration throughout the experiment.

### Conditioned value learning

**FIGURE 1:**
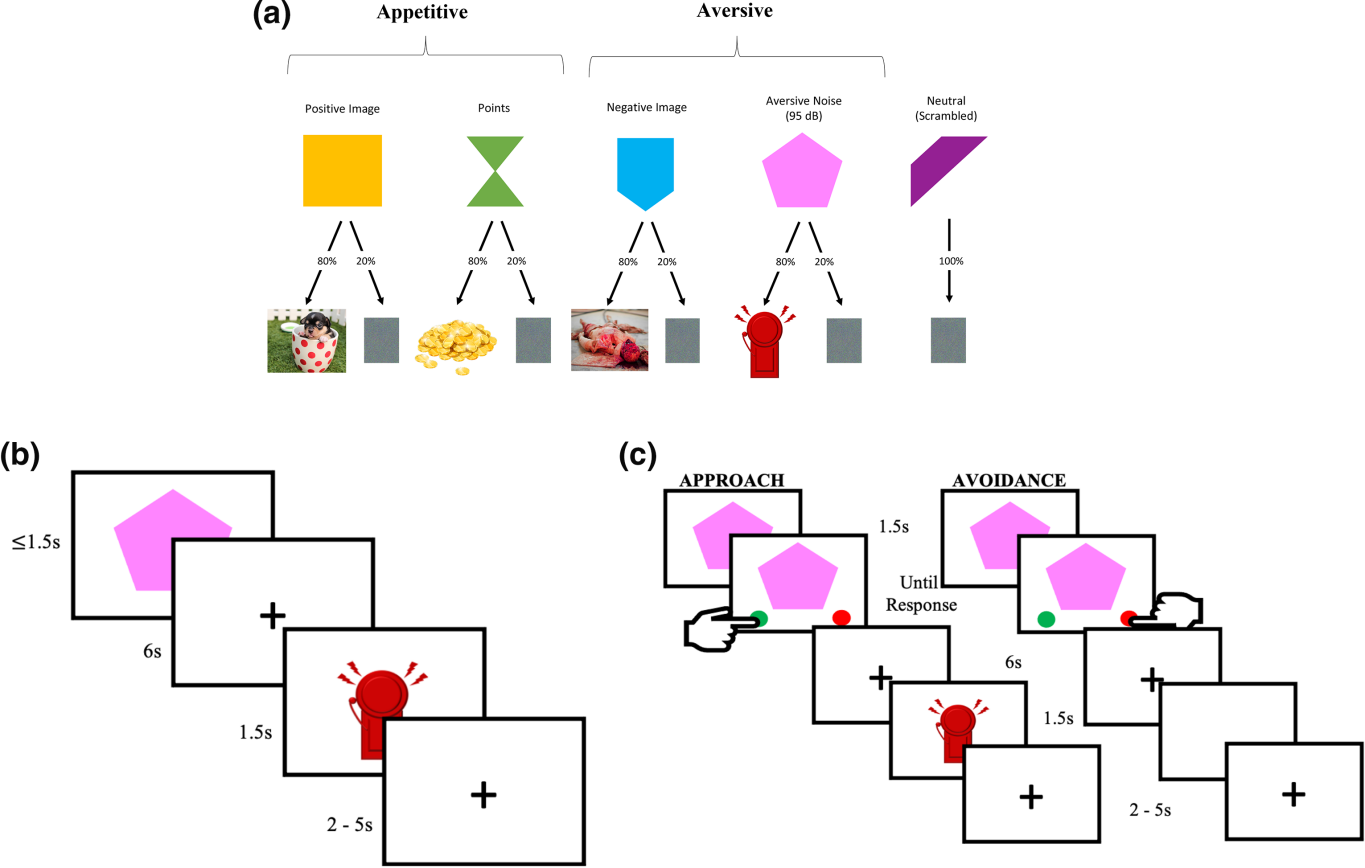
Task schematics. (a) Example of pairings between colored shapes and appetitive, aversive, and neutral outcomes. Pairings were counterbalanced across subjects. (b) In the conditioned learning task, children saw a colored shape followed by a valued outcome. (c) In the behavioral choice task, children made decisions about whether to approach or avoid presentation of the stimuli that were previously associated with valued outcomes during conditioning

Participants completed a Pavlovian conditioning paradigm (Metereau & Dreher, 2015) in which they were exposed to five colored shapes, each of which was followed by an appetitive, aversive, or neutral reinforcer (Figure 1). Appetitive reinforcers were points earned and a positive image; aversive reinforcers were an unpleasant 95 dB noise and a negative image. The images were taken from the Open Affective Standardized Image Set (OASIS; Kurdi et al., 2017; Positive Image: I256; Negative Image: I287). We used a variety of different reinforcer types to assess whether findings generalize across different reinforcers. During conditioning, participants saw a visual cue (geometric colored shape) that was displayed until a keyboard response was made or 1.5 s had passed. After this cue, there was a delay period of 6 s during which a fixation cross was displayed. Next, either a corresponding reinforcer or a scrambled neutral image appeared for 1.5 s with a probability of 0.8 for the reinforcer and 0.2 for the scrambled neutral image. Each trial was followed by a jittered inter‐trial interval of 2.5–5.5 s. A fifth neutral condition consisted of a geometric cue that was always paired with a neutral scrambled picture. To maintain attention and as a measure of conditioning, participants were asked to press a keyboard response button as soon as they saw the geometric cue. Participants completed 14 trials of each condition for a total of 70 trials. Presentation of each trial was randomized within participants, and the shape‐reinforcer pairings were fully counterbalanced using a Latin Square design across participants.

To measure learning, participants rated how good or bad they thought each colored shape stimuli was prior to and after the conditioning task using a Visual Analogue Scale (VAS) that ranged from 0 (bad) to 100 (good) (Figure S1). Response times (RTs) were also used to model participants’ learning rates using a Rescorla and Wagner reinforcement learning framework (1972). In this reinforcement learning framework, learning occurs through updating expectations in proportion to prediction errors (or the discrepancy between expected outcomes and actual outcomes) so that across trials the expected outcome value converges to the actual outcome value. We derived learning rates using RTs to the colored shape stimuli, which have been demonstrated to be good indicator of conditioning (Critchley et al., 2002; Gottfried et al., 2003); learning rates represent the speed of integration of recent outcomes (Glimcher, 2011; Nussenbaum & Hartley, 2019).

As a tertiary convergent measure of conditioned value learning, we also examined heart rate reactivity. Heart rate was derived from ECG data recorded continuously throughout the study and were analyzed as inter‐beat intervals of the heart (IBI) sampled at 4 Hz. IBI represents the time in milliseconds between two heart beats; as heart rate decreases, IBI increases. ECG was measured and analyzed using a Bionex system (MindWare Technologies LTD). To examine whether there were differences in heart rate reactivity during anticipation and presentation of reinforcers, IBIs were coded for the 6‐s anticipatory period between cue presentation and reinforcer presentation to assess reactivity in anticipation of the reinforcer. IBIs were also coded for the time period between reinforcer presentation and next cue presentation to assess autonomic reactivity to the reinforcers (4–7 s). We used IBIs which are the measure of reactivity as they are conducive to examining changes in autonomic reactivity on short time scales (Berntson et al., 2007; Dimitroff et al., 2017).

### Use of learned information to guide behavioral choice

After the conditioned learning task, participants completed a behavioral choice task in which they used value information from the conditioning task to decide whether to approach or avoid appetitive and aversive stimuli (Figure 1). Participants were presented with the same shapes they encountered on the previous task. After 1.5 s, a green and a red button appeared on either side of the screen. These buttons remained on screen until participants made a response. If participants selected the green button, the trial proceeded as in the conditioning task—the paired reinforcer was presented. However, if participants selected the red button, a blank screen appeared without any reinforcer. In this manner, selecting the green button represented an approach response and pressing the red button represented an avoidance response. As in the conditioning task, participants completed 14 trials of each condition for a total of 70 trials. Trial presentation was randomized within participants and the side of the screen where the green and red buttons appeared was counterbalanced across participants.

### Measures of early life stress and perceived social isolation

To determine if stressful life events and perceived social isolation were associated with performance on the conditioned learning or behavioral choice tasks, we measured both children's stressful event exposures by having parents completed the Coddington Life Events Checklist (Coddington, 1972). On this checklist, parents indicated whether children had been exposed to any of a set of 40 potentially stressful life events (see Table S1), with responses summed. We chose to use this checklist as it was designed to capture a broad range in the type and severity of potentially stressful events. We measured children's perceptions of social isolation using the Loneliness and Social Dissatisfaction Questionnaire (Cassidy & Asher, 1992). Both measures have demonstrated good reliability and validity. In the current sample, both measures had high internal consistency (Loneliness and Social Dissatisfaction Questionnaire: *α* = .91; Coddington Life Events Checklist: *α* = .74).

### Memory of learned information

To ensure that task performance did not reflect participants forgetting the shape‐reinforcer pairings, children completed an explicit recall task. Memory was assessed two different ways. In one block, participants saw each colored shape and were asked to identify what came after it by selecting one of four choices. In another block, participants were presented with each reinforcer and asked to identify what came before it by selecting one of four choices. Presentation of trials within blocks was randomized, and order of blocks was counterbalanced across participants. Details of the memory task are shown in Figure S1.

### Statistical analyses

We used hierarchical linear modeling (HLM; lmer and glmer functions in the lme4 package in R v4.0.5; Bates et al., 2015; R Core Team, 2019) to test the associations between early life stress, perceived social isolation, and children's performance on the conditioned learning and behavioral choice tasks. All models included a participant level random intercept. Children's stressful event exposures, perceived social isolation, and an interaction between children's event exposures and perceived social isolation were included as fixed participant level predictors. All continuous predictors were standardized. Significance of all fixed effects was assessed using the ANOVA function in the car package (Fox & Weisberg, 2019). To control for multiple comparisons in our primary analyses, *p*‐values were false discovery rate (FDR) corrected within each model for the number of model terms (Benjamini et al., 2001). The emmeans package (Lenth, 2019) to examine simple slopes for interactions in linear models as recommended by Preacher et al. (2006) and estimated marginal effects for predicted response probabilities for interactions in logistic models as recommended by Long and Mustillo (2021). Children's perceived social isolation scores ranged from 16 to 65 (*M* = 28.94, *SD* = 10.07), and children's stressful event exposures ranged from 0 to 20 (*M* = 7.42, *SD* = 4.24). The two measures were not correlated (*r* = .16, *p* = .18). One participant was missing data for the behavioral tasks due to technological failures and one missing data for stressful event exposures leaving a total of 70 participants included in the final analyses. Three additional participants were missing IBI data due to moving artifacts or noise, leaving a total of 68 participants for analyses of heart rate reactivity. One additional participant was missing memory data leaving a total of 69 participants for memory analyses. Further methodological and analytical details are presented in Supporting Information.

## RESULTS

### Early life stress, perceived social isolation, and conditioned value learning

Children effectively learned the associated pairings as assessed by multiple indices of learning including changes in children's VAS ratings of the colored shape stimuli (*χ* ^2^(4) = 15.98, *p* = .024) and their heart rate reactivity during anticipation of the reinforcer (*χ* ^2^(4) = 47.75, *p* < .001; Table 1). Across the sample, the best fit learning rate was 0.2 which is similar to those utilized in other studies (Jensen et al., 2007; Metereau & Dreher, 2015; O’Doherty et al., 2006), providing additional evidence children learned the paired associations. We did not find evidence that children's stressful event exposures and perceptions of social isolation affected their ability to learn value information. First, we examined differences in participants’ pre‐ and post‐conditioning ratings of the colored shape stimuli using a 3‐level HLM model. This analysis included a random intercept for reinforcer type (points, positive image, aversive noise, negative image), with reinforcer type nested within participant. Rating time (pre, post‐conditioning) was included as a fixed factor nested within reinforcer type. There were no effects of stressful event exposures (*χ* ^2^(4) = 4.01, *p* = .818), perceived social isolation (*χ* ^2^(4) = 0.49, *p* = .974), or interactions between stressful event exposures and perceived social isolation (*χ* ^2^(4) = 2.59, *p* = .817) on changes in children's ratings of stimuli after conditioning. We confirmed this finding through two additional analyses. There was also no relation between stressful event exposures (*χ* ^2^(1) = 0.74, *p* = .812), perceived social isolation (*χ* ^2^(1) = 0.01, *p* = .933), and learning rates (Interaction: *χ* ^2^(1) = 0.98, *p* = .812), nor did heart rate reactivity reveal evidence of stressful event exposures (*χ* ^2^(4) = 1.90, *p* = .946) or perceived social isolation (*χ* ^2^(4) = 1.09, *p* = .946) on learning (Interaction: *χ* ^2^(4) = 2.67, *p* = .946). Reported *p*‐values for VAS ratings were FDR corrected for 16 comparisons, for learning rates eight comparisons, and for heart rate 32 comparisons (number of model terms). Controlling for age, gender, household income, parental education, depressive and anxiety symptoms, and general cognitive ability did not change any of the reported effects for our primary measure of learning.

**TABLE 1:** Simple slopes for Visual Analogue Ratings and IBI reactivity during conditioning task

Outcome	Reinforcer type	*β* (*SE*)
Visual Analogue Ratings	Neutral	5.38 (3.42)
	Points	5.46 (3.42)
	Positive image	1.69 (3.42)
	Aversive noise	−10.38 (3.42)
	Negative image	5.51 (3.42)
IBI reactivity	Neutral	−15.40 (4.78)
	Points	−16.30 (4.66)
	Positive image	−19.10 (4.61)
	Aversive noise	15.60 (4.78)
	Negative image	−28.00 (4.66)

Note

For Visual Analogue Ratings slope represents post‐rating–pre‐rating. For inter‐beat intervals (IBIs) slope represents anticipation–stimulus.

### Early life stress, perceived social isolation, and behavioral choice

**FIGURE 2:**
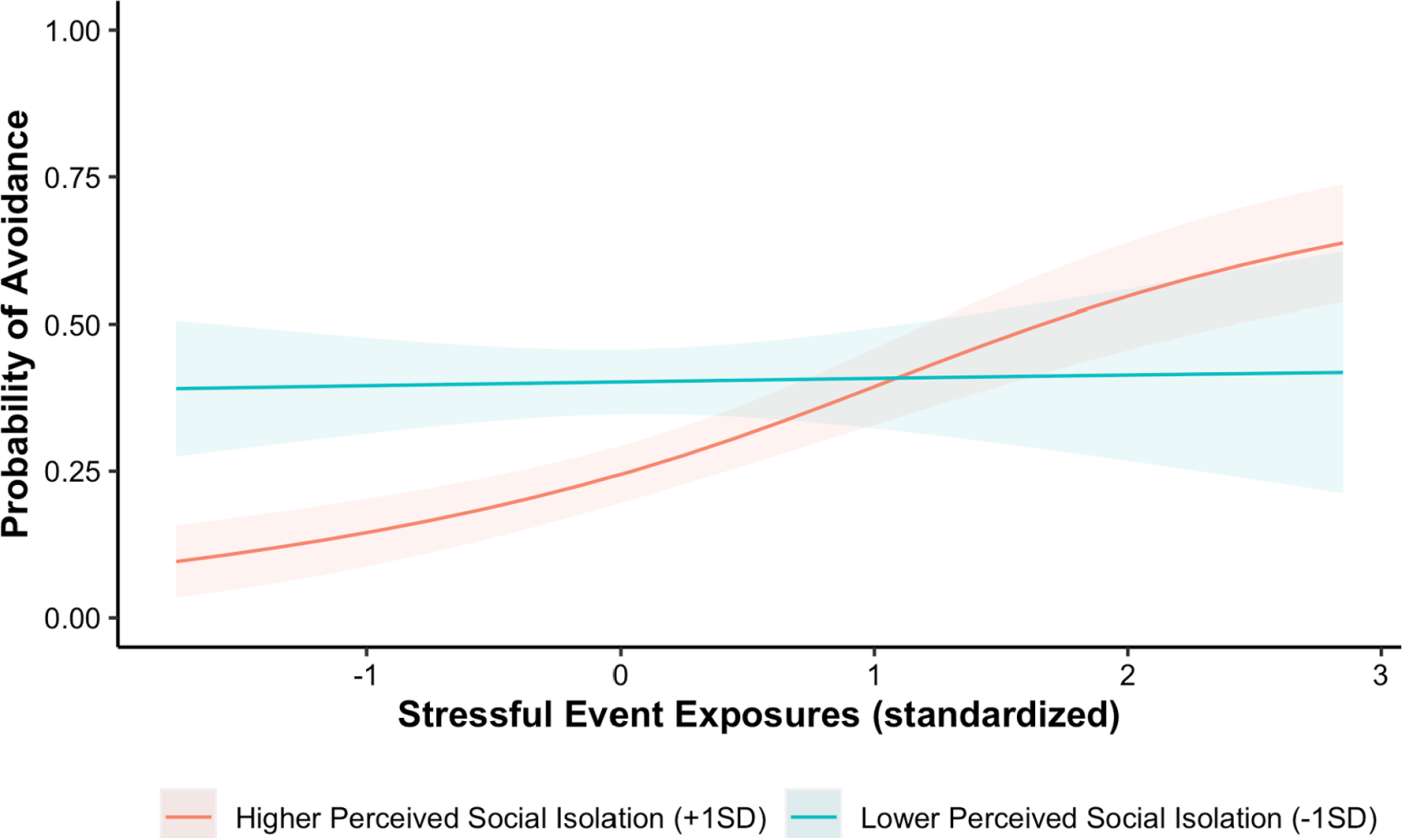
Effects of stressful life event exposure and perceived social isolation on children's avoidance behaviors. Stressful life event exposure was positively associated with increased likelihood of avoidance (*β* = 0.25, *SE* = 0.08, *p* = .003) as compared to approach of reinforcers for children with higher levels of perceived social isolation (in red). There was no relation between stressful life event exposure and children's avoidance behaviors (*β* = 0.01, *SE* = 0.13, *p* = .921) for those reporting lower levels of perceived social isolation (in blue).

In contrast to conditioned value learning, we did find associations between stressful event exposures, perceptions of social isolation, and children's performance on the behavioral choice task (Figure 2a). We ran a 3‐level logistic multilevel model with a random intercept for reinforcer type, and reinforcer type nested within participant. Whether or not a child chose to avoid the reinforcer was the outcome. *p*‐Values were FDR corrected for eight comparisons (number of model terms). There were effects of both stressful event exposures (*χ* ^2^(1) = 8.49, *p* = .014) and perceptions of social isolation (*χ* ^2^(1) = 7.08, *p* = .021) on children's behaviors. Children with more stressful event exposures were more likely to avoid (*β* = 0.15, *SE* = 0.07, *p* = .035) both positive and negative reinforcers. The effect of perceived social isolation perceptions was in the opposite direction, with higher levels of perceived social isolation being associated with decreased probability of avoidance behaviors (*β* = −0.16, *SE* = 0.08, *p* = .040).

Of primary interest to our hypothesis, there was an interaction between stressful event exposures and perceptions of social isolation (*χ* ^2^(1) = 4.65, *p* = .049). Children with more stressful event exposures were more likely to avoid (*β* = 0.25, *SE* = 0.08, *p* = .003) only if they reported higher levels of perceived social isolation. Children with lower levels of perceived social isolation demonstrated no differences in how they avoided reinforcers (*β* = 0.01, *SE* = 0.13, *p* = .921), regardless of their exposure to stressful events. Controlling for age, gender, household income, parental education, depressive and anxiety symptoms, and general cognitive ability did not change any of the reported effects.

To rule out the possibility that performance on the approach and avoidance task was due to differences in how children attended during the task, we examined whether children's stressful event exposures and perceptions of social isolation were associated with differences in reaction times for their responses on the behavioral choice task. There were no associations between stressful event exposures (*χ* ^2^(1) = 2.81, *p* = .299), perceived social isolation (*χ* ^2^(1) = 0.15, *p* = .879), or interactions between the two (*χ* ^2^(1) = 0.29, *p* = .869) and children's reaction times. Additionally, to rule out the possibility performance differences were due to children forgetting the paired associations, we tested whether children's experiences of stress predicted their recall of the shape‐reinforcer pairings. There were no associations between stressful event exposures (*χ* ^2^(1) = 0.26, *p* = .812) or perceived social isolation (*χ* ^2^(1) = 0.81, *p* = .737) and memory for reinforcer pairings (Interaction: *χ* ^2^(1) = 7.15, *p* = .060). *p*‐Values for analyses with reaction times were FDR corrected for 16 comparisons and for recall were FDR corrected for eight comparisons (number of model terms).

## DISCUSSION

This study tested the relation between stressful event exposures, perceptions of social isolation, and children's ability to both learn about and benefit from salient cues in their environments. We sought to overcome limitations of prior research in two ways. First, we used separate measures to examine children's ability to learn valued outcomes as well as their ability to use that information to adaptively guide their behaviors. Second, we included measures of children's exposure to potentially stressful events along with their perceptions of social isolation, which have been linked to increased perceptions of stress and hypersensitivity to environmental threat (Brown et al., 2017; McHugh & Lawlor, 2013; Smith et al., 2020). We did not find consistent evidence that early life stress or perceived social isolation were related to children's ability to learn pairings between cues and valued reinforcers. However, both children's stressful event exposures and perceived social isolation were associated with their ability to use learned information to guide their behaviors. Specifically, stressful event exposures were related to children's use of learned information among youth who also perceived themselves to be socially isolated. In contrast, there was no association between stressful event exposures and use of learned information to guide behavior for children who reported lower levels of perceived social isolation.

The present data suggest that the effects of stress on how children use value information are not driven solely by events in their lives. Rather, these effects emerge among children who report they perceive themselves to lack high‐quality social relationships. One potential explanation for these effects is that children who perceive themselves to be socially isolated may be more likely to construe events in their environments as stressful. This is in line with research from adults and non‐human animals suggesting that perceived social isolation exacerbates psychological and biological responses to stress, placing individuals at greater risk for long‐term negative stress‐related outcomes (Brown et al., 2017; Hawkley & Capitanio, 2015). Additionally, it parallels with research indicating individual variability in stress and stress‐related perceptions, not events, determines long‐term biobehavioral outcomes (McEwen & Akil, 2020; Smith & Pollak, 2020). These findings suggest that assessment of children's perceptions and interpretations of their environment, in addition to their event exposures, will better elucidate the mechanisms underlying variability in children's outcomes after stress. However, there are two possible alternative explanations for these data. One is that children perceiving themselves to be socially isolated actually experienced more stressful events. This explanation has little empirical support given that these two measures were uncorrelated in the present sample. Another possibility is that highly stressed children simply failed to encode the stimulus‐outcome pairings or attended more poorly during the task. This explanation is also not supported by data in that neither stress exposure nor perceived social isolation was related to memory for the pairings or reaction times during the task. Future research, assessing perceptions of social isolation along with child reported exposure to stressful events and perceptions of stress, can provide further insight into the mechanism through which perceptions of social isolation influence children's perceptions of event in their environment.

Prior research has proposed two potential explanations for the relation between early life stress and altered performance on associative learning tasks. The first is that children with high‐stress exposure are less able to recognize and learn probabilistic associations in their environment (Gerin et al., 2017; Harms et al., 2018). Alternatively, altered performance could be indicative of alterations in the mechanisms which translate learned information into action (Birn et al., 2017; Dillon et al., 2009). Yet, prior research has not utilized approaches that allow these two processes to be examined separately. The current findings provide evidence for the latter—childhood stress appears to disrupt the translation of learned information into behavior. Additionally, the present observation of increased avoidance among children with high‐stress exposure and high perceived social isolation in this sample aligns with both evidence for habitual avoidance responding (Patterson et al., 2019) as well as reports of increased sensitivity to threat cues (Briggs‐Gowan et al., 2015; Shackman & Pollak, 2014) in children exposed to early childhood stress. The present data also support recent proposals that the effects of early life stress on neural processing during reward learning are linked to deficits in children's approach motivations rather than learning (Novick et al., 2018). The specificity of the effects of childhood stress on the translation of learned information into action, rather than learning itself, likely support motivational goals aimed at prioritizing survival—children in environments perceived as highly threatening learn contingent relations but use that knowledge to prioritize avoiding threats. Indeed, shifting behaviors towards those aimed at avoiding threats, even if it results in the loss of potential rewards or other resources, would represent an adaptive strategy in a high threat environment. It is possible other aspects of early stressful environments, like predictability, may have more specific effects on value learning. Research directly assessing predictability (both environmental and perceived) can inform this question.

Of note, we find that perceived social isolation in the context of low stressful event exposure is associated with increased approach behaviors rather than increased avoidance. One potential explanation for this is that we did not target recruitment towards children with high levels of stressful event exposure or social isolation. This means we may have been indexing more acute levels of perceived social isolation. In rodents, there is evidence that post‐weaning social isolation increases appetitive approach behaviors (Cuenya et al., 2015; Hong et al., 2012; Jahng et al., 2012), and research with humans finds that acute social exclusion increases social approach behaviors (Maner et al., 2007; Van Roekel et al., 2014; Wesselmann et al., 2012). In contrast, chronic social isolation, has been demonstrated to increase social withdrawal and avoidance behaviors (Hawkley & Capitanio, 2015; Qualter et al., 2015; Vanhalst et al., 2018). Further exploration of the relation between acute and chronic perceptions of social isolation and approach and avoidance motivations can aid in elaborating the mechanisms underlying this relation.

An outstanding issue related to understanding the effects of early life stress on value‐based decision making is how to conceptualize early life stress. Prior research has focused on children who have been exposed to poverty or maltreatment (Herzberg & Gunnar, 2020; Novick et al., 2018) while this study examined a broader range of potentially stressful life events in a normative sample of children which may limit comparability. However, recent debate over how to best conceptualize and measure stress in childhood (Pollak & Smith, 2021; Richter‐Levin & Sandi, 2021; Smith & Pollak, 2020) indicates a need for more research assessing multiple different aspects of the environment along with children's perceptions to better understand what contributes to experiences of stress and adversity. There is a dearth of research examining how the event‐based measures we typically use to assess stress in childhood are associated with children's perceptions and experiences of stress. Research taking a broader approach looking at a wider range of factors across different samples of children is critical to better understanding what contributes to variability in children's stress‐related outcomes. Indeed, recent research using a machine learning approach across a large sample of children found little relation between standard measures of the family and environment, including measures of poverty and potential maltreatment, and children's later outcomes (Salganik et al., 2020). For this reason, we chose to utilize a broad life events measure of potential stressors in combination with measures of stress‐related perceptions, specifically perceptions of social isolation. Future research can examine these questions in samples typically considered more normative and samples typically considered high stress, along with measures of timing of event exposures and perceived severity, to better understand how different aspects of the early environment contribute to children's value learning.

The current study assessed value learning within a limited age range of 8–9 years. We chose this age range as it is one in which there is reliable evidence for both threat and reward learning. But it is possible that there could be differences in a younger sample or an adolescent sample, as both reward and threat learning change during development (Galván, 2013; Michalska et al., 2016). Future research can examine how the relation between early life stress, perceived social isolation, and value learning may change over the course of development. Additionally, our study focused on perceptions that have been implicated as critical to shaping stress responses, those of social isolation, but did not directly measure children's perceptions of discrete stressful events. Given growing research suggests children's perceptions of potentially stressful events play a key role in shaping their long‐term developmental outcomes (Allwood et al., 2017; Danese & Widom, 2020), future research examining children's perceptions of specific event exposures could provide additional insight into how stress in childhood influences value learning. Last, in the current study, we did not target recruitment towards children at high risk for stress exposure but examined variation in stressful event exposures and perceived social isolation in a normative sample that was primarily White, within a small age range, and of relatively high socioeconomic status. This along with the smaller sample size somewhat limits the generalizability of our findings. Future research can explore whether these relations differ in larger samples of children exposed to particularly high levels of stressful life events, across different age ranges, and in children of different socioeconomic, ethnic, and cultural backgrounds.

Overall, these data cast a new light on the relation between early life stress and a critical component of learning in children. They suggest that early life stress does not necessarily influence how children learn about value relations in their environment, but rather shifts how they prioritize the information when informing behaviors. This altered weighting of value information may explain links between early stress and behaviors viewed as maladaptive or “problem” behaviors, like impulsivity or aggression. This study also highlights the importance of incorporating factors beyond children's event exposures in models about the mechanisms through which early life experiences can affect the development of brain‐behavior relations. Research aimed at further examining the factors that alter the way learned information is used in decision making holds potential for informing effective interventions for youth at high risk for behavioral and educational problems.

## CONFLICT OF INTEREST

The authors have no conflicts of interests to declare.

## Supporting information

Supplementary Material
