# Supplementary material for: Early life stress and perceived social isolation influence how children use value information to guide behavior
Source: Child Dev. 2021 Dec 31;93(3):804–14. doi: 10.1111/cdev.13727 (PMC9177517; doi:10.1111/cdev.13727)
Supplement: Supplementary file 1 — Supplementary Material [file CDEV-93-804-s001.docx]

Supplemental Materials

Additional Methodological Details

Conditioned Learning Task

Appetitive and aversive images were rated similarly for arousal (Appetitive Image: I256, M_valence_ = 6.49, SD_valence_ = 0.78, M_arousal_ = 5.03, SD_arousal_ = 1.72; Aversive Image: I287, M_valence_ = 1.16, SD_valence_ = 0.42, M_arousal_ = 5.15, SD_arousal_ = 2.34). Points rewards and the aversive noise were accompanied by both visual and auditory presentations: the points were conveyed by an image of a pile of coins and the sound of a cash register, and the aversive noise involved presentation of an alarm bell and the sound of an alarm buzzer. To attach value to the points reward, children were told that the type of prize they received would depend on the number of points they earned in the game. Adults were told they could receive additional money dependent on the number of points earned (up to $5). At the end of the study, child participants were allowed to choose any prize and adults received the full dollar amount regardless of performance.

Computational Model and Additional Measures of Learning

We used a Rescorla-Wagner rule (Rescorla & Wagner, 1972) to model learning for each condition. For each trial *t* a prediction error δ(t) was computed as the difference between the actual outcome value R(t) and it’s expected value V(T) on that trial (Eq (1)):

$$\delta\left( t \right)=R\left( t \right)-V(t)$$

Then, the expected value of the next trial V(t + 1) was updated by adding the prediction error δ(t) weighted by a learning rate α (Eq. (2)).

$$V\left( t+1 \right)=V\left( t \right)+ \alpha\delta$$

The outcome value R(t) was set to 1 when a reinforcer was delivered and to 0 when a scrambled picture was displayed. V(t) was initialized to 0. The learning rate, α, represents the speed of integration of recent outcomes (Glimcher, 2011; Nussenbaum & Hartley, 2019).

We derived participant level learning rates using subjects’ response times (RTs) to the cue using participants’ keyboard responses to neutral shapes during the conditioning task. RTs have been shown to be good indicators of conditioning (Critchley, Mathias, & Dolan, 2002; Gottfried, O’Doherty, & Dolan, 2003) and correlated with the prediction V(t) estimated by reinforcement learning models (Seymour et al., 2004). We derived the prediction V(t) for each participant based on their individual conditioning histories for a range of learning rates (from 0 to 0.5). We then fit trial by trial RTs to a regression model that included the prediction V(t) for each modeled learning and compared regression fits to determine which learning rate best fit participants’ behavior. Across participants the best fit yielded a learning rate of 0.2 which is similar to those utilized in other studies (Jensen et al., 2007; Metereau & Dreher, 2015; O’Doherty, Buchanan, Seymour, & Dolan, 2006). RTs were in milliseconds and log-transformed to allow analysis across subjects. In line with previous research, we excluded outlier trials with RTs greater than 3 standard deviations from each subjects mean RT (Otto & Daw, 2019). Using participant level learning rates, we then modeled participant level expected value for each trial to identify participants maximum expected value associated with each neutral shape.

Statistical Analyses

We used HLM techniques to examine the relationship between experiences of stress and children’s conditioned learning, behavioral choice, and explicit recall. All models included a random intercept for participant and stressful life event exposures and perceived social isolation were included as fixed participant level factors. For models assessing children’s learning rates, reinforcer type was included as a fixed factor nested within participant. In models examining children’s heart rate reactivity, time epoch in task, interval (anticipation of reinforcer and reinforcer presentation), and reinforcer type (points, appetitive image, aversive noise, negative image, and neutral) were included as fixed factors nested within subject. A random slope was also included for time epoch in the task.

Each participant’s performance on the explicit recall task was measured using: *hit rate* (HR) which refers to the probability of selecting the target; *false alarm rate* (FAR), which refers to the probability of selecting a response which did not match the target. HRs and FARs were combined into a statistic (*d’*) that describes an individual’s sensitivity to targets.

*d'* = z(HR) – z(FAR)

For the discrimination index, an HLM model was run including a fixed effect for reinforcer type nested within participant to assess effects of experiences of stress on explicit recall.

**Additional Effects**

Learning in Conditioning Task

Examining the simple slopes for the interaction effect between reinforcer type and rating time indicated that children demonstrated increases in ratings of the conditioned stimuli paired with the points (β = 5.46, SE = 3.42, p = 0.112) and positive image (β = 1.69, SE = 3.42, p = 0.622) and decreases in ratings for conditioned stimuli paired with the aversive noise (β = -10.38, SE = 3.42, p = 0.003). However, children demonstrated increases in ratings for the negative image (β = 5.51, SE = 3.42, p = 0.108).

In further support that children learned the associated pairings there were interactions between interval (anticipation/presentation) and reinforcer (χ^2^(4) = 47.75, p < 0.001), and interval, reinforcer type and time (χ^2^(4) = 19.81, p = 0.003). The interaction between interval and reinforcer type appeared to be driven by the points and aversive noise demonstrating smaller differences in IBIs between anticipation and reinforcer presentation (Table 1). Additionally, examining the simple slopes for the interaction between interval, reinforcer type, and time in task indicated that over the course of the task, IBI reactivity during anticipation decreased for the aversive noise, becoming more similar to reactivity to the reinforcer at the start of the task. Similarly, IBI reactivity for the points and positive images showed less decrease over the course of the task during anticipation suggesting maintenance of IBI reactivity similar to that during reinforcer presentation for the points and positive images (Table S2). Together this is indicative of IBI reactivity during anticipation of the reinforcer, particularly for the points and aversive noise, becoming more similar to that during reinforcer presentation suggestive of learning of paired associations. While heart rate is multiply influenced by a number of psychological processes, these effects, in concert with the behavioral findings, suggest children learned the shape-reinforcer pairings, particularly those for the points and aversive noise.

Controlling for age, gender, household income, parental education, depressive and anxiety symptoms, and general cognitive ability did not change any of the reported effects for visual analogue scale ratings and heart rate. However, it did result in the main effect of loneliness on learning rates becoming significant (*χ*^2^(1) = 7.24, *p* = 0.007) such that children with higher levels of loneliness demonstrated smaller learning rates (*β* = -0.10, SE = 0.04, p = 0.007) indicative of faster integrative of information.

Table S1. Frequencies of reported stressful events

| Event | Percentage Reporting Experienced |
| --- | --- |
| Addition of sibling | 37.5 |
| Death grandparent | 31.9 |
| Marital separation parents | 20.8 |
| Parent loss of job | 23.6 |
| Moved home | 56.9 |
| Increase in arguments with parents | 37.5 |
| Decrease in arguments with parents | 25.0 |
| Increase in income | 31.9 |
| Change in parents’ occupation increasing parent absence | 25.0 |
| Outstanding personal achievement by child | 52.8 |
| Serious illness of sibling | 8.3 |
| Mother beginning work | 33.3 |
| Change of school | 50.0 |
| Sibling leaving home | 5.6 |
| Change in peer relationships | 11.1 |
| Serious illness requiring hospitalization of sibling | 2.8 |
| Increase in parental arguments | 25.0 |
| Decrease in parental arguments | 23.6 |
| Serious illness requiring child hospitalization | 5.6 |
| Accident causing concussion or serious injury of child | 15.3 |
| Death of friend of child | 0.0 |
| Addition of adult to family | 9.7 |
| Discovery of being adopted | 1.4 |
| Parent in jail | 1.4 |
| Death of parent | 0.0 |
| Divorce of parents | 12.5 |
| Child acquires visible deformity | 0.0 |
| Death of sibling | 1.4 |
| Marriage of parent to step-parent | 2.8 |
| Parent with alcohol or drug problem | 5.6 |
| Mother pregnant | 31.9 |
| Change in parent’s health status | 11.1 |
| Death of a pet | 38.9 |
| Death of family member or friend | 15.3 |
| Family victim of crime | 4.2 |
| Increase in arguments between child and siblings | 36.1 |
| Parent mental health issue | 18.0 |
| Child having trouble at with teachers | 6.9 |
| Child failed a grade | 0.0 |
| Other | 15.3 |

Table S2. Simple slopes between reinforcer type, interval, and time for IBIs

| **Reinforcer Type** | **Interval** | **β (SE)** | **p** |
| --- | --- | --- | --- |
| Neutral | Anticipation | -0.26 (0.06) | < 0.001 |
|  | Reinforcer | -0.33 (0.06) | < 0.001 |
| Points | Anticipation | -0.15 (0.06) | 0.006 |
|  | Reinforcer | -0.29 (0.06) | < 0.001 |
| Positive Image | Anticipation | -0.19 (0.06) | 0.001 |
|  | Reinforcer | -0.27 (0.06) | < 0.001 |
| Aversive Noise | Anticipation | -0.28 (0.06) | < 0.001 |
|  | Reinforcer | -0.09 (0.06) | 0.127 |
| Negative Image | Anticipation | -0.27 (0.06) | < 0.001 |
|  | Reinforcer | -0.38 (0.06) | < 0.001 |

Figure S1*.* Procedure for Pre- and Post-conditioning Ratings and Explicit Recall Task

*Note.* A) Procedure for pre- and post-conditioning ratings of neutral shapes using Visual Analogue Scale (VAS) ranging from bad (0) to good (100). Participants were asked to respond to “How good or bad is this” by clicking on the gray scale bar. B) Procedure for explicit recall task. This task consisted of two blocks, counterbalanced across participants. In one block, participants saw the shape for 1.5 seconds and were asked to identify what came after the shape. In the other block, participants saw the reinforcer and were asked to identify what shape came before it.


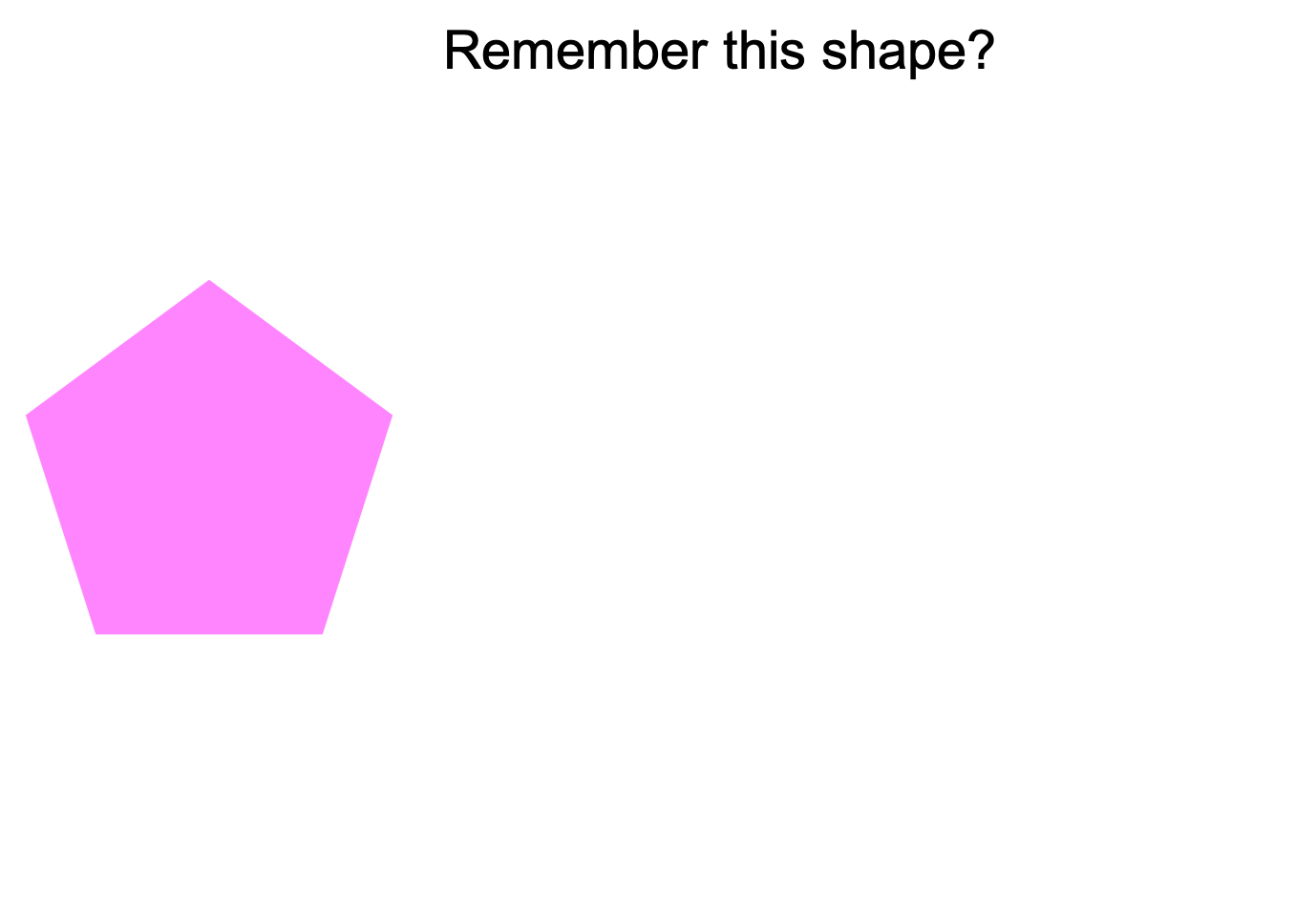


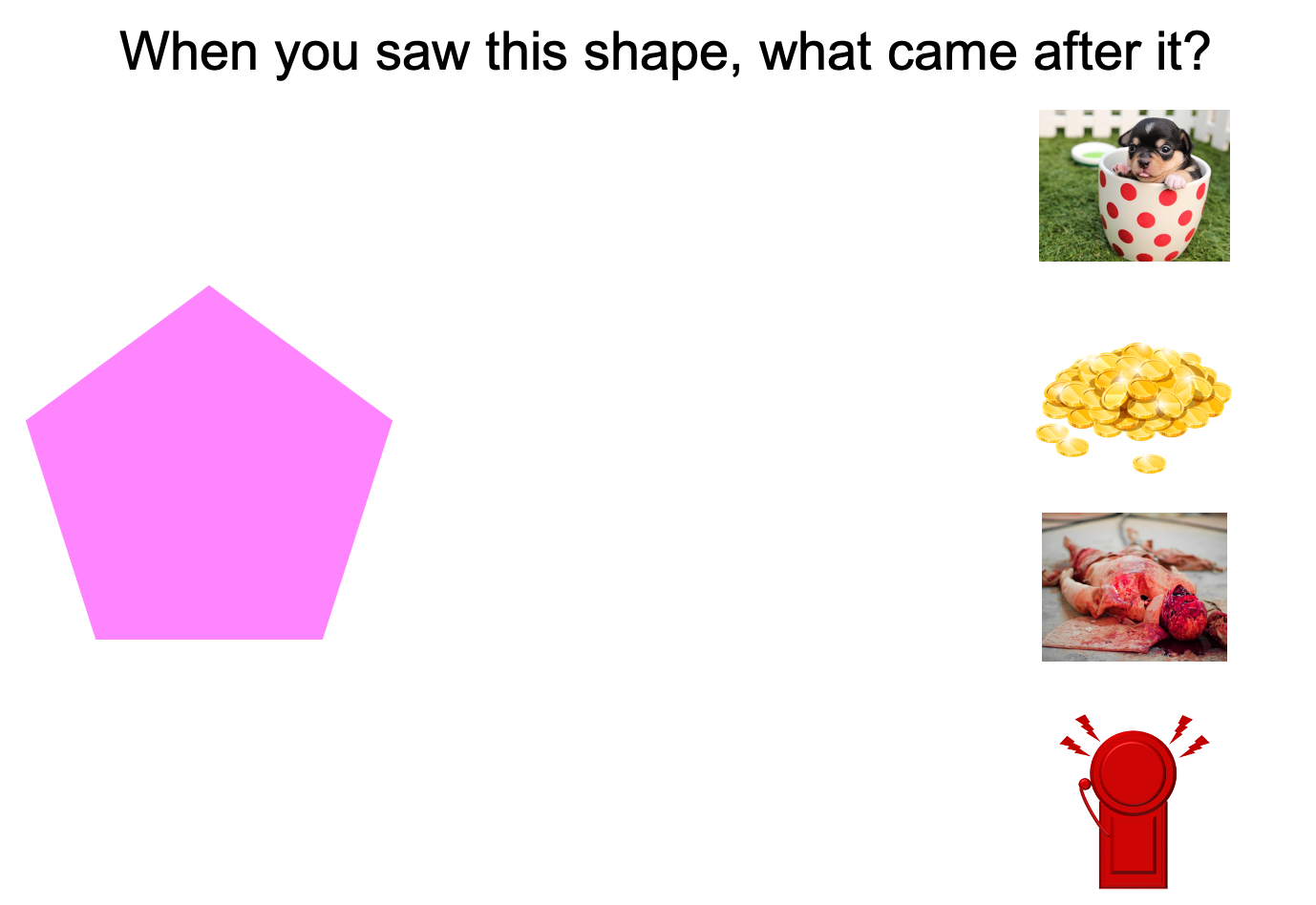


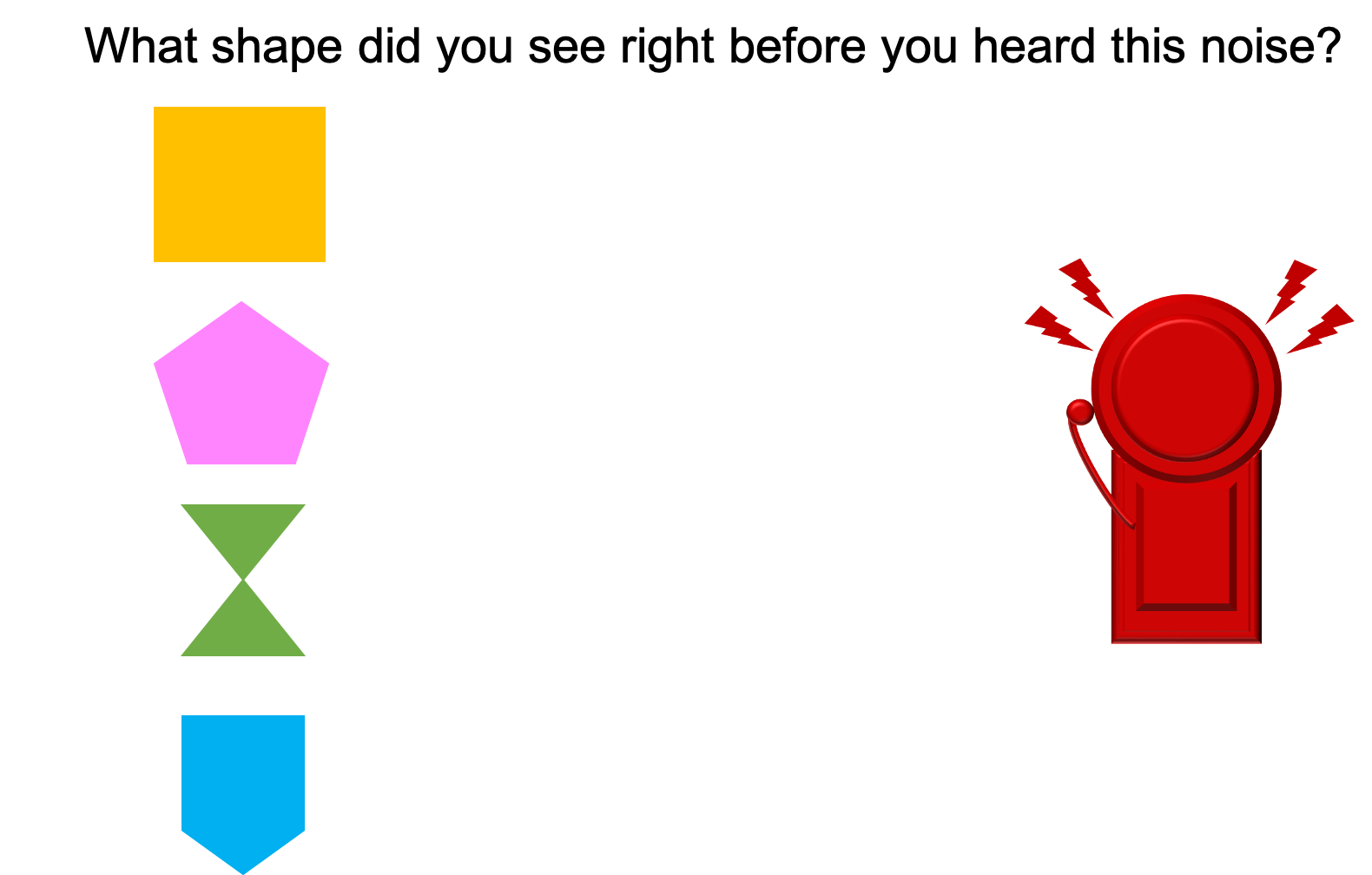


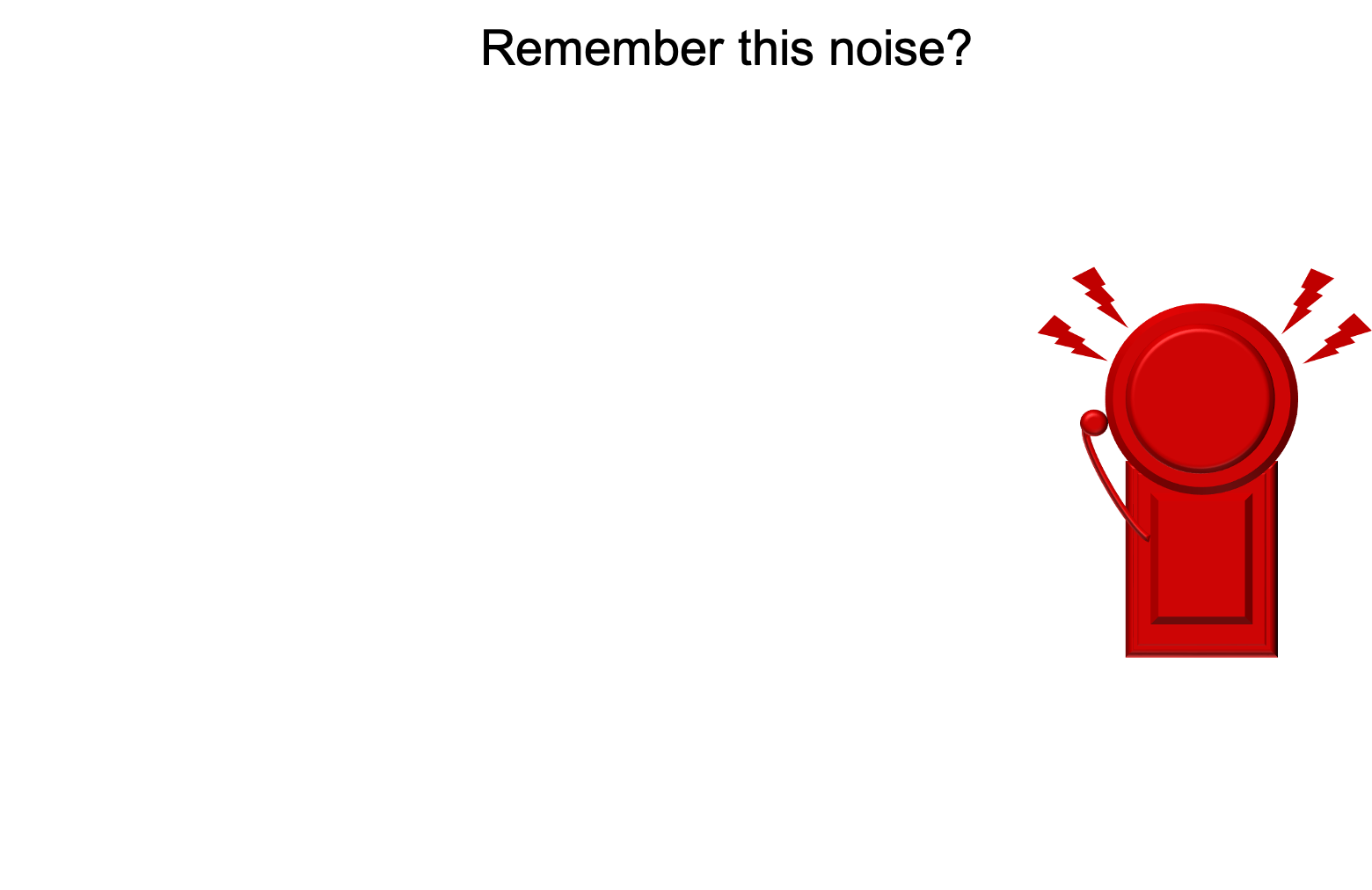


A)

B)

1.5s

1.5s


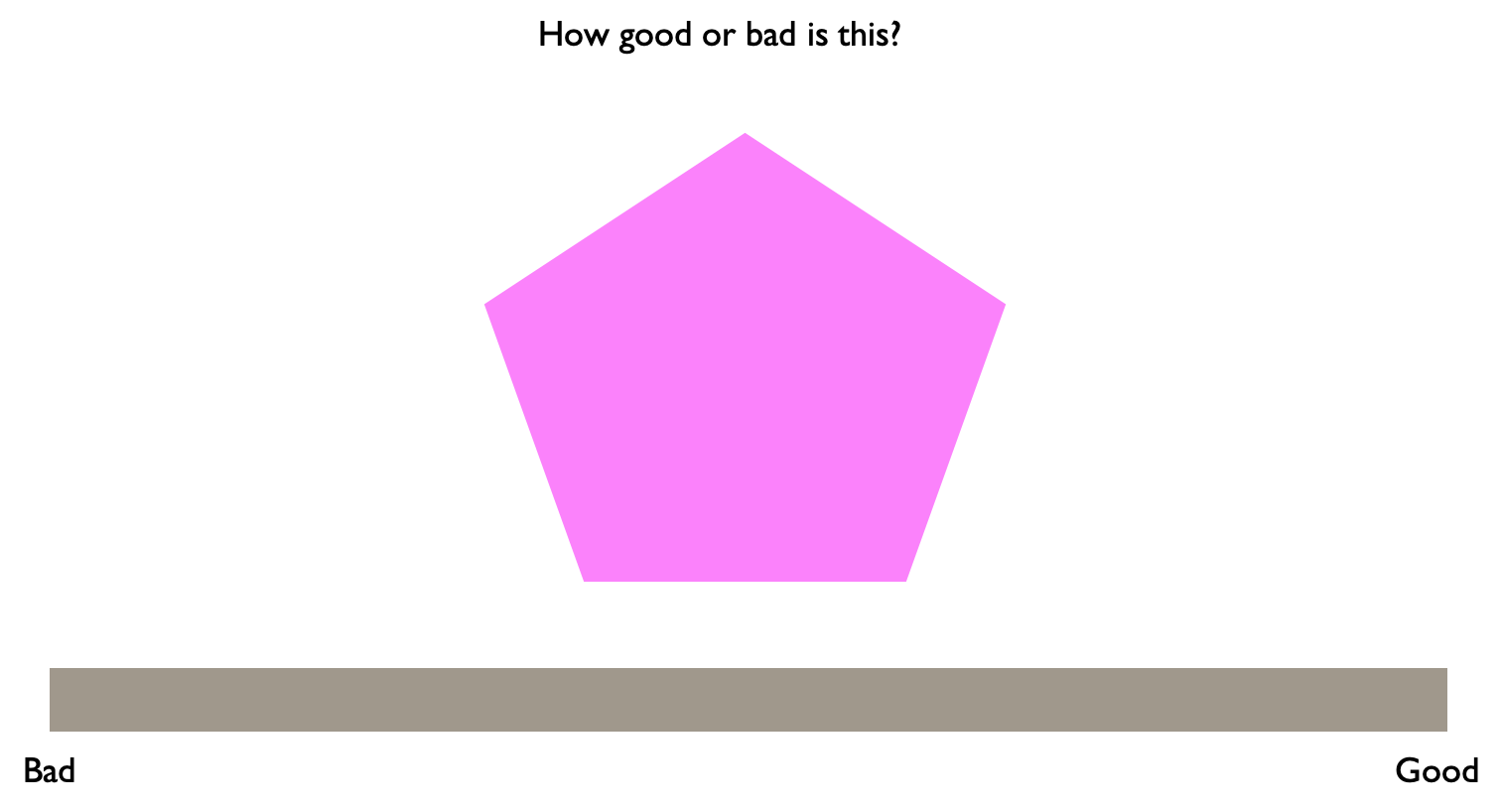


Figure S2*.* Interaction between stressful event exposures and perceived loneliness for behavior choice by condition


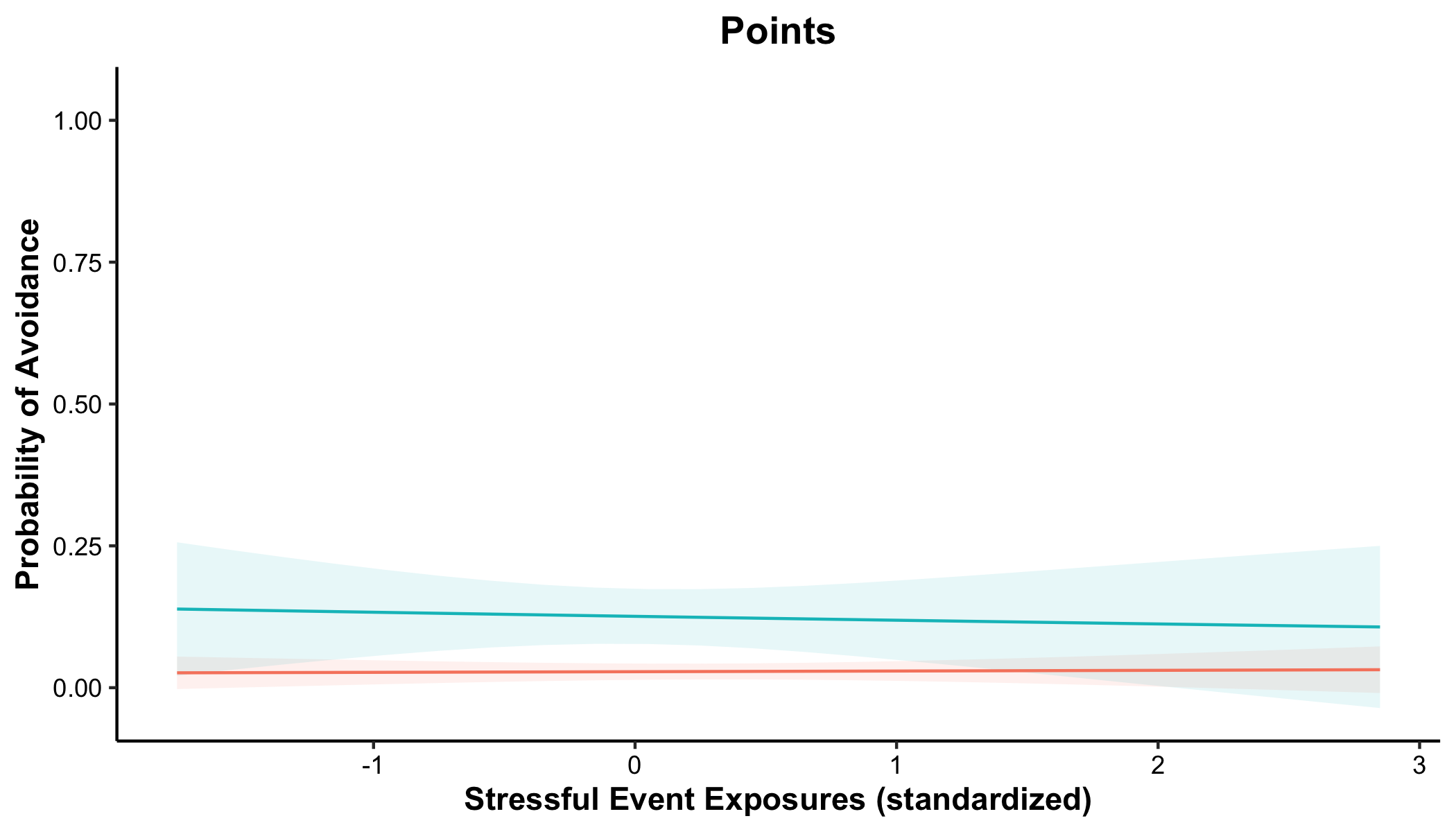


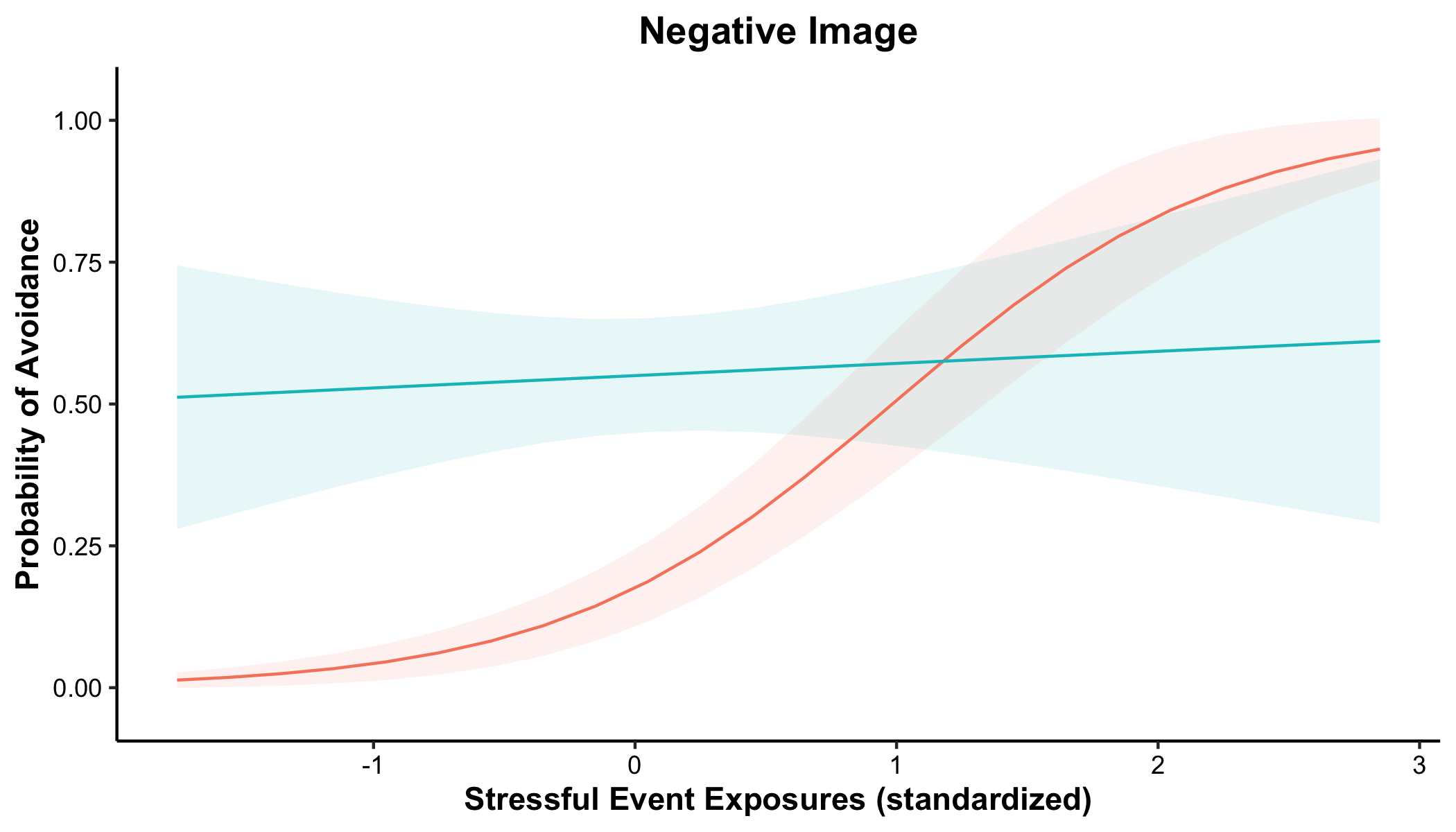


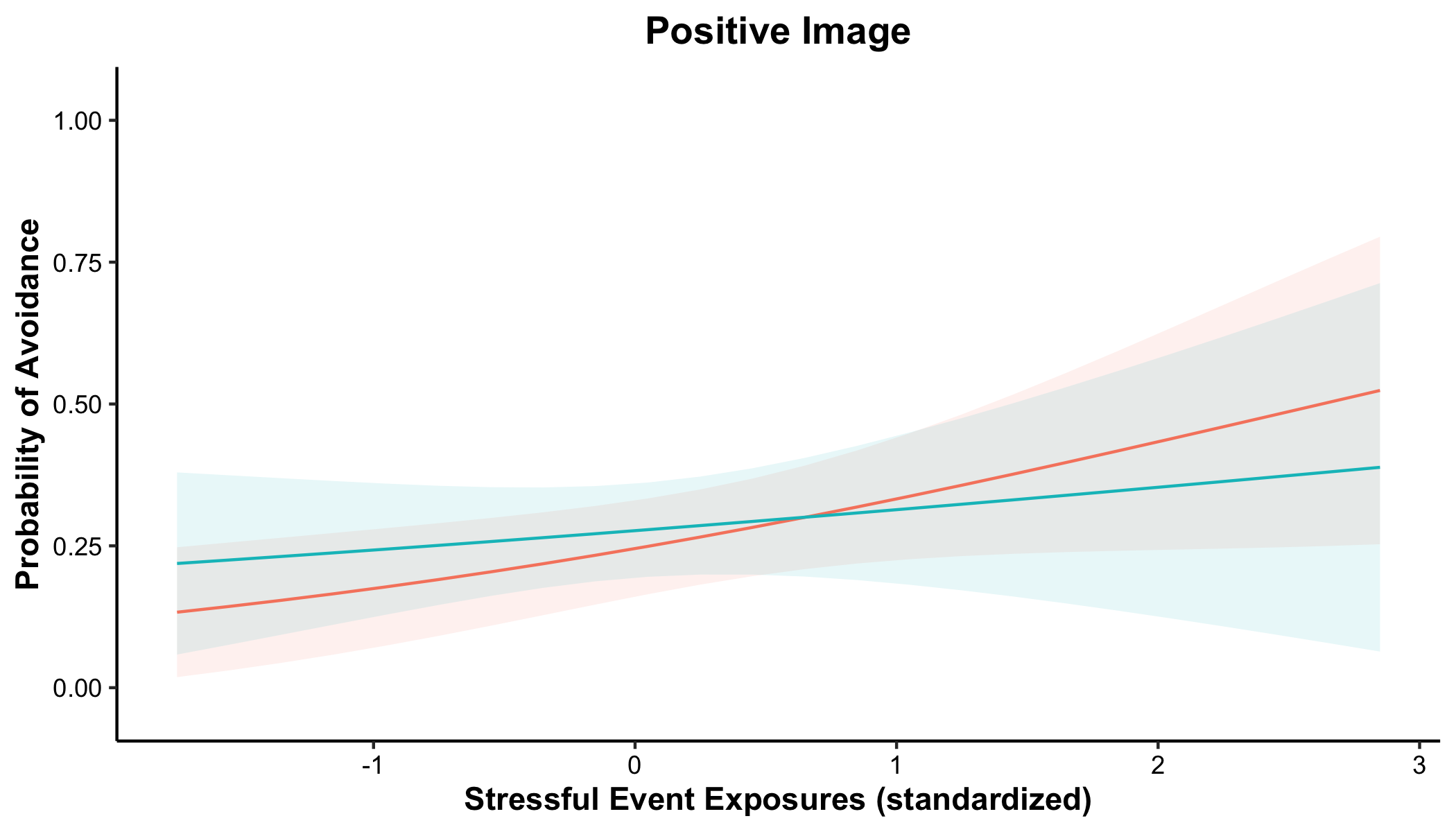


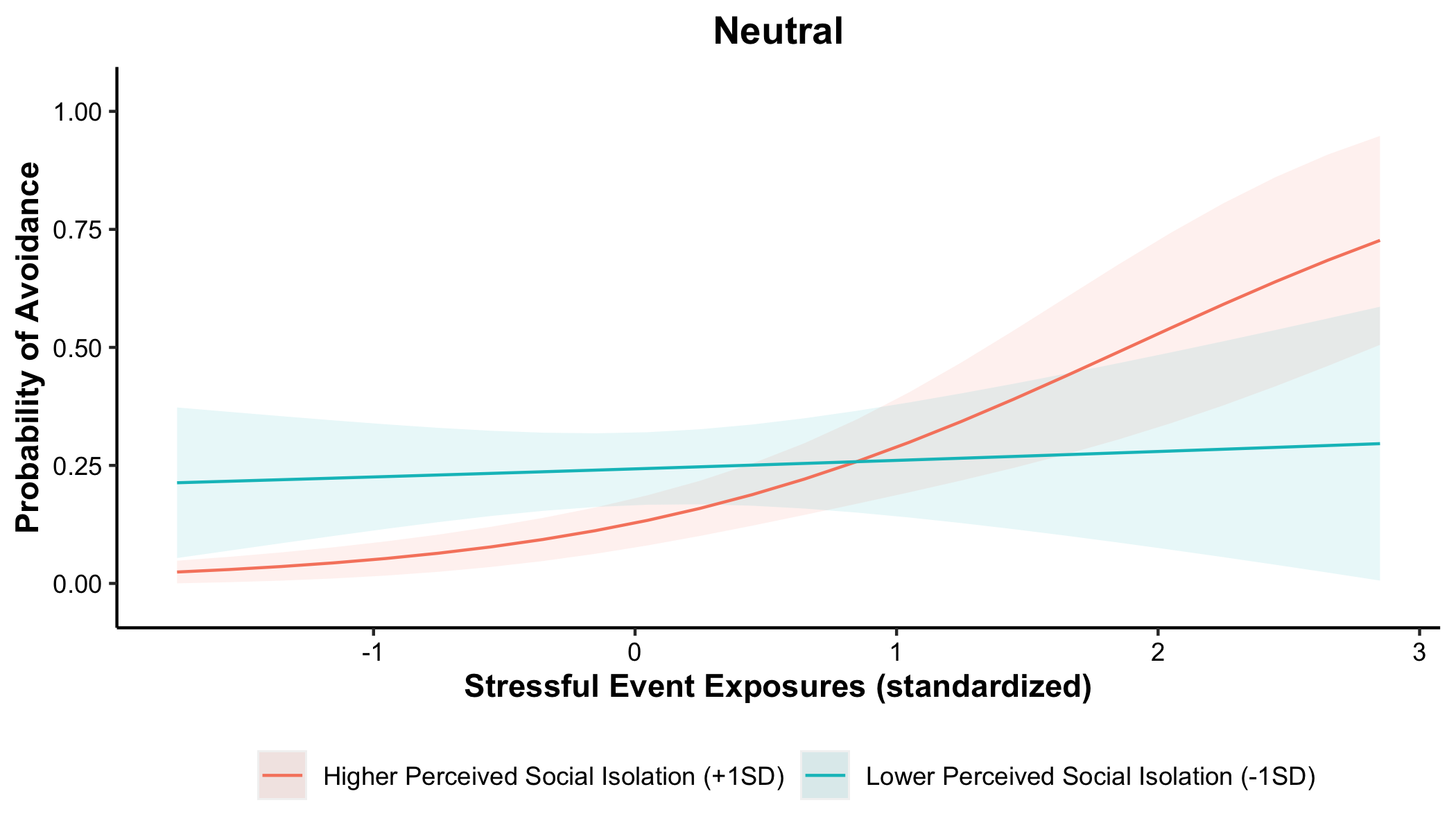


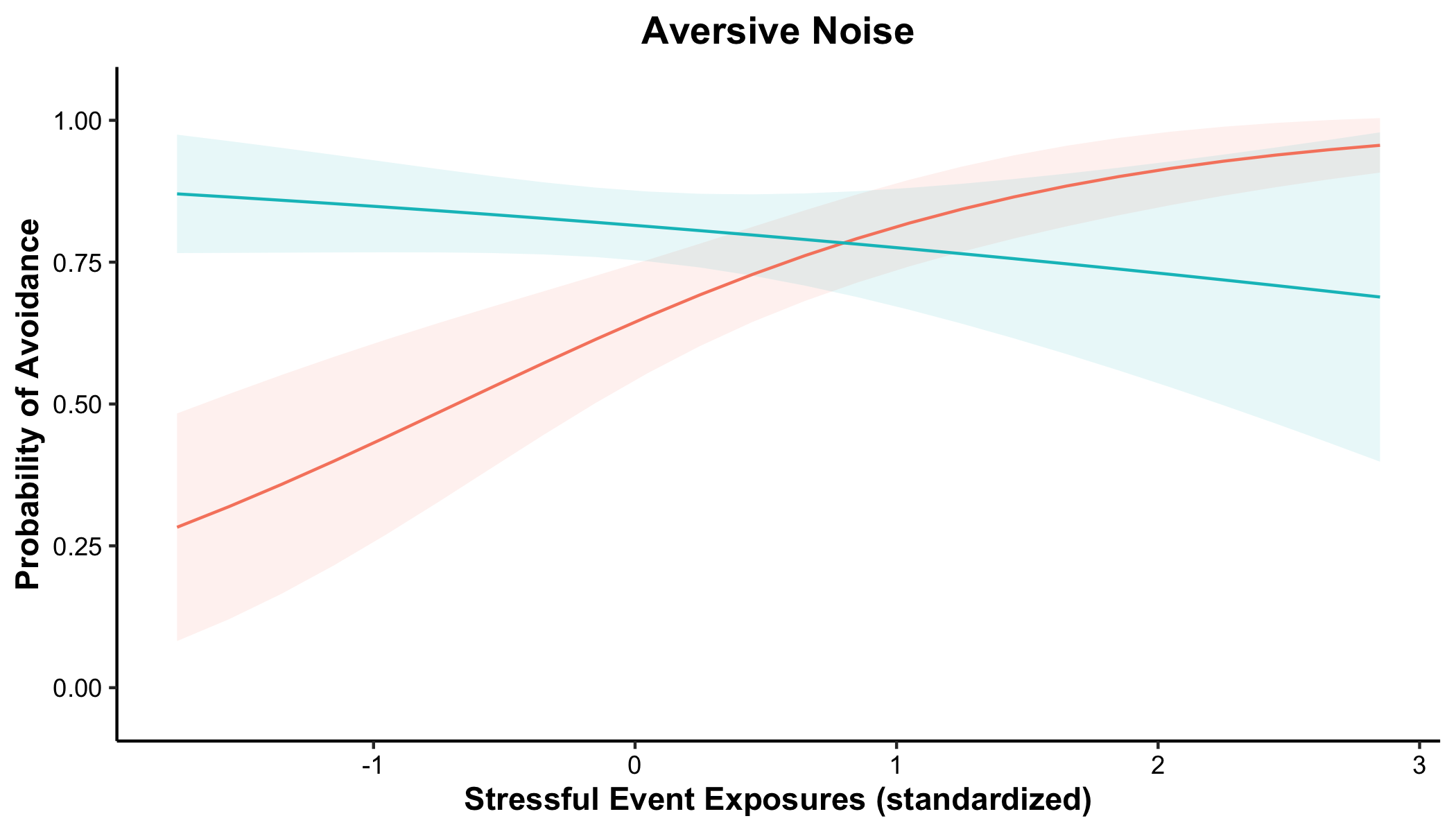


*Note.* Avoidance behaviors by reinforcer condition. While there was no significant interaction between stress event exposures, perceived social isolation, and reinforcer condition (χ^2^(4) = 4.61, p = 0.329), these graphs are included for interested readers.
